# Effect of a mobile phone-based interactive voice response on common childhood illnesses in Ghana: a quasi-experimental study

**DOI:** 10.1186/s12978-025-01985-4

**Published:** 2025-05-31

**Authors:** Princess R. Acheampong, Aliyu Mohammed, Sampson Twumasi-Ankrah, Augustina A. Sylverken, Michael Owusu, Timothy K. Adjei, Emmanuel Acquah-Gyan, Ellis Owusu-Dabo

**Affiliations:** 1 Department of Health Promotion and Disability Studies, School of Public Health, College of Health Sciences, Kwame Nkrumah University of Science and Technology, Kumasi, Ghana; 2 Department of Epidemiology and Biostatistics, School of Public Health, College of Health Sciences, Kwame Nkrumah University of Science and Technology, Kumasi, Ghana; 3 Department of Statistics and Actuarial Science, College of Science, Kwame Nkrumah University of Science and Technology, Kumasi, Ghana; 4 Department of Theoretical and Applied Biology, College of Science, Kwame Nkrumah University of Science and Technology, Kumasi, Ghana; 5 Department of Medical Laboratory Technology, Kwame Nkrumah University of Science and Technology, Kumasi, Ghana; 6 Department of Global and International Health, School of Public Health, College of Health Sciences, Kwame Nkrumah University of Science and Technology, Kumasi, Ghana

**Keywords:** Mobile health, Childhood illnesses, Malaria, Diarrhoea, Cough, Efficacy, Implementation, Accessibility, Health education, Activated voice response

## Abstract

**Background:**

Malaria, acute respiratory infections (ARIs), and diarrhoea are primary causes of morbidity and mortality among children under five years old in Ghana. Despite the implementation of various interventions, the nation struggles to meet relevant health and policy targets. While the potential of mobile health interventions to enhance child health outcomes has been recognized, their impact on prevalent childhood illnesses remains insufficiently explored. This implementation research study aimed to evaluate the effect of a mobile health information system (mHIS) intervention on common childhood illnesses among under-five children residing in rural health districts of Ghana.

**Methods:**

In this quasi-experimental study, we enrolled all children under five years old from randomly selected clusters within the rural intervention and control health districts in the Ashanti region, Ghana between November 2018 and December 2021. The Reach, Effectiveness, Adoption Implementation and Maintenance (RE-AIM) framework was used to design and implement the intervention. The intervention involved a mobile phone-based information system to monitor childhood conditions, offer telemedicine consultations, and deliver child health promotion messages on nutrition and management of common childhood illnesses to caregivers. By employing the average treatment effect (ATET) and difference-in-difference (DiD) analyses, we assessed outcome disparities in diarrhoea, cough, and presumptive malaria.

**Results:**

The incidence of diarrhoea and malaria decreased in the intervention group. The ATET analysis indicated pre-intervention disparities in presumptive malaria with a post-intervention difference between the groups for diarrhoea and presumptive malaria. Results related to cough, used as a proxy for ARIs, did not provide conclusive results across the intervention and control sites based on this intervention. However, the DiD model highlighted an overall statistically significant reduction in diarrhoea and presumptive malaria.

**Conclusion:**

This study underscores the effectiveness of a mobile phone-based health information system intervention in curbing common childhood morbidities, particularly diarrhoea and presumptive malaria, among under-five children in rural Ghana. This approach demonstrates promise in advancing child health outcomes and contributing to the reduction of prevalent illnesses in resource-constrained settings.

## Résumé

**Contexte:**

Le paludisme, les (infection aiguë des voies respiratoires (IAVR)et la diarrhée sont les principales causes de morbidité et de mortalité chez les enfants de moins de cinq ans au Ghana. Malgré la mise en œuvre de diverses interventions, le pays peine à atteindre les objectifs pertinents en matière de santé et de politique. Bien que l’on ait reconnu le potentiel des interventions de santé mobiles pour améliorer les résultats en matière de santé des enfants, leur impact sur les maladies infantiles prévalentes n’a pas encore été suffisamment exploré. Cette étude de recherche de mise en œuvre visait à évaluer l’effet d’une intervention de système d’information sanitaire mobile (mSGIS) sur les maladies infantiles courantes chez les enfants de moins de cinq ans résidant dans les districts sanitaires ruraux du Ghana.

**Méthodes:**

Dans cette étude quasi expérimentale, nous avons recruté tous les enfants de moins de cinq ans issus de groupes sélectionnés au hasard dans les districts sanitaires ruraux d’intervention et de contrôle de la région d’Ashanti, au Ghana, entre novembre 2018 et décembre 2021. Le cadre de portée, d’efficacité, d’adoption, de mise en œuvre et de maintenance (RE-AIM) a été utilisé pour concevoir l’intervention et la mettre en œuvre. L’intervention consistait en un système d’information sur téléphone mobile pour surveiller l’état de l’enfant, offrir des consultations de télémédecine et diffuser aux soignants des messages de promotion de la santé infantile sur la nutrition et la prise en charge des maladies infantiles courantes. En utilisant les analyses de l’effet moyen de traitement (ATET) et de la différence dans la différence (DiD), nous avons évalué les disparités de résultats en matière de diarrhée, de toux et de paludisme présumé.

**Résultats:**

L’incidence de la diarrhée et du paludisme a diminué dans le groupe d’intervention. L’analyse de l’ATET a révélé des disparités avant l’intervention dans le paludisme présumé avec une différence après l’intervention entre les groupes pour la diarrhée et le paludisme présumé. Les résultats liés à la toux, utilisée comme indicateur des IAVR, n’ont pas fourni de résultats concluants dans l’ensemble des sites d’intervention et de contrôle sur la base de cette intervention. Cependant, le modèle DiD a mis en évidence une réduction globale statistiquement significative de la diarrhée et du paludisme présumé.

**Conclusion:**

Cette étude souligne l’efficacité de l’utilisation d’un système d’information sanitaire par téléphone mobile pour réduire les morbidités infantiles courantes, en particulier la diarrhée et le paludisme présumé, chez les enfants de moins de cinq ans dans les zones rurales du Ghana. Cette approche s’avère prometteuse pour améliorer les résultats en matière de santé infantile et contribuer à la réduction des maladies prévalentes dans les milieux où les ressources sont limitées.

## Introduction

Every child has the inherent right to life, but sadly, an estimated 5.9 million children across the globe die before the age of five [1]. Sub-Saharan Africa bears the highest under-5 mortality rate in the world, as it contributes to 50% of the estimated global under-5 deaths [2]. It is estimated that one out of every 13 children die before the age of five [3]. In Ghana, statistics show that 1 out of every 17 children die before their fifth birthday, as reported by the Ghana Demographic and Health Survey [4]. The Ghana Under-5 Child Health Policy from 2007 to 2015 was implemented to reduce under-5 mortality from 60 deaths per 1000 live births to 40 deaths per 1000 live births by 2015 [5]. Although this ratio decreased at the end of the national policy to 45 deaths per 1000 live births in 2020 [5], several gaps remain.

Despite several interventions, policies and strategies such as the National Health Insurance Scheme (NHIS), and Community-based Health and Planning and Services (CHPS) that have also been rolled out, Ghana is still reported to be among eight countries that are making very little progress in the reduction of under-5 mortality in Africa [4]. Unfortunately, the majority of these under-5 deaths are caused by easily manageable or preventable diseases such as malaria, acute respiratory infections (ARI), measles, and diarrheal diseases (or a combination of such diseases), with malnutrition playing a significant role in the under-5 mortality burden [6].

Delays in seeking healthcare have been documented to have grave consequences on reducing the under-5 mortality burden in rural communities in Ghana and other countries in the sub-region [7]. Several studies conducted elsewhere show that distance from the health care facilities, poor knowledge about the symptoms of diseases, perceived in-curability of illness, lack of money, limited health care access, and a long period of waiting for medical services were the main reasons for low health care utilization in developing countries [8].

Mobile health (mHealth) technology, which is still in its early stages and relies on communication and mobile phone technologies in healthcare, has emerged as a possible game changer [9]. Interactive voice response (IVR) systems, have emerged as a possible mHealth option for providing health information, encouraging adherence to treatment standards, and improving health outcomes [9–12]. Mobile phone-based IVR offers the potential to decrease some of the conventional obstacles to health education and service delivery in Ghana.

Mobile phone use in Ghana has expanded rapidly over the past two decades and has become an essential part of everyday life for many people. The country has seen substantial growth in mobile phone penetration, driven by the increasing availability of affordable mobile devices and improvements in network infrastructure. The uptake of mHealth in Ghana is influenced by a complex interplay of social determinants of health. Socioeconomic status, education, geographic location, access to technology, and social norms all play a role in determining how and to what extent mHealth solutions are adopted and utilized. The uptake of mHealth solutions can however, these solutions are always embedded within existing social and digital determinants of health [12].

Using an implementation research framing, we assessed the effect of a mobile phone voice response in lowering the frequency of selected common paediatric illnesses (malaria, diarrhoea and cough), on the incidence of under-five morbidity and mortality. Our mobile phone interactive voice response was designed and implemented using the RE-AIM framework which considers the intervention's Reach, Effectiveness, Adoption, Implementation, and Maintenance. By situating this study within the RE-AIM framework, we designed and implemented the effect of mHealth interventions on child health outcomes in rural Ghana.

Reach focuses on the extent to which the intervention engages the target population, and in this study, we targeted legal parents or guardians of children under five within an 8 km radius of a health facility, ensuring accessibility to those most at risk. Effectiveness examines the intervention's impact on health outcomes, and we assessed the reduction in the incidence of diarrhoea, cough, and malaria by comparing baseline and endline data. Adoption involves the uptake of the intervention by stakeholders, and we engaged District Health Management Teams, opinion leaders, and caregivers in developing culturally appropriate health education messages to foster community support and participation. Implementation refers to the fidelity of the intervention delivery, and we disseminated health information via voice SMS every two weeks to ensure consistent and reliable communication, thereby improving health knowledge and practices among caregivers. Maintenance assesses the sustainability of the intervention's effects, and we evaluated the effect of the mHealth intervention over a 30-month period to determine its potential for enduring benefits.

## Methods and materials

### Study design and setting

We conducted an interventional study designed to assess the effect of a mobile phone-based health information system (mHIS) capable of harnessing self-reports and disseminating health information in the form of voice—using an automated voice response system- to caregivers of children under-five. This study was named, MobChild, to indicate the tool (mobile phone) and the target group (child under-5).

All messages were disseminated in the local dialect, Twi. A quasi-experimental design was employed to compare an intervention group and control group with respect to the incidence of common under-5 childhood illnesses such as diarrhoea, cough and malaria. Cough was selected as one of the childhood illnesses because it serves as a proxy to ARIs in children under-5.

Cross-sectional surveys were conducted every 6 months for 3 years, from caregivers of children under-5 residing in two separate health districts from November, 2018 to October, 2021. The intervention district, Asante Akyim North district (Coordinate: 6°19′53.1″N 1°02′56.5″W) and the control district (Coordinates: 6°37′5″N 1°12′36″W) are located in the Ashanti Region of Ghana. The population of both districts mainly engage in small-scale farming. The Agogo Presbyterian Hospital (APH) and Juaso District Hospital serve as referral health facilities and serve as referral centres for other hospitals in the region. Some areas in the two districts are virtually inaccessible during the rainy season. Telephone services are available in both districts except in very remote towns. Most of the roads leading to these towns are inaccessible to motorists. Thus, these communities are served by the smallest health unit (CHPS) and referred to hospitals where necessary. These districts were selected based on a previous pilot study in the area [13].

### Study population

The population of interest were:

- Legal parents or guardians (caregivers) of children aged 0–59 months with access to a mobile phone and residing within 8 km radius of a health facility. The maximum distance of these communities from the referral hospitals was approximately 40 km.
- All children aged 0–59 months (newborns to five-year-olds) within the study areas. Once a child turned five during the period, they were excluded.

### The intervention

Telephone numbers obtained from caregivers during recruitment were uploaded onto a mobile phone-based health information system (mHIS) used to implement the intervention. The IVR component of the system was based on the established clinical algorithm (Integrated Management of Childhood Illnesses, (=IMCI). The use of the IMCI implied answering a set of questions and collecting data on current symptoms [13]. The MobChild IVR system can be used notwithstanding the type of phone, either smart or unsmart.

The content of the health education messages was developed after wide stakeholder consultation with the District Health Management Teams, opinion leaders and caregivers. The content of the health messages, disseminated every two weeks, was designed to improve the health knowledge of the caregivers and motivate them to engage in healthy practices in their local language (Twi). An example of the messages was, “*If your child experiences persistent fever for more than 2 weeks consult a physician immediately*.” To maintain the interest of the caregivers, the content of the messages usually started with a drama, and this varied on three themes; general knowledge about childhood illnesses, danger signs and preventive measures. The scheduled messages were automatically disseminated as bulk messages and re-sent to users who did not receive them, repeated every two hours a day for two (2) days. In total, 35,075 voice SMS were successfully delivered to caregivers in the intervention group over the 30-month intervention period. Caregivers received all messages for free.

The MobChild IVR system, also called the mHis system, was designed to allow a caller to dial into the computer system over a telephone line and access a service running on the computer. The caller may then interact with and receive voice information from the service. All calls were free for caregivers. The mHIS system was designed to collect health data and provide tailor-made health information to its users even in remote areas where internet access is absent and at the same time automatically disseminate health information in the form of voice SMS to registered users. Users had the opportunity of calling a health hotline to describe the disease symptoms of their sick child in order to obtain information whether to seek professional care or whether it is fine to treat their children at home.

### Sample size and sampling technique

Based on a previous study [8] that showed a response rate of 23.4%, an estimated 1200 caregivers and 1300 children under-5, were recruited to participate in the study with a default alpha of 5% and power of 80%.

A two-stage cluster sampling design was employed to select the participants in each arm. The first sampling stage involved the selection of a representative number of clusters from the four sub-districts, with a fair representation of communities located in the remote areas in the sampling frame. A total of 23/115 and 40/200 clusters, based on the Ghana 2010 Population and Housing Census, were randomly selected for the intervention and control sites, respectively. The total clusters required for each study arm were based on proportional sampling.

All eligible households (households with children under-5) members within the selected clusters were included in the study. All eligible caregivers (caregivers with children under-5) and their children under-5 were recruited to participate in the study after securing informed consent. The selected clusters were in an 8 km acceptable range to the health facility. All health facilities within this radius that serve the selected clusters were involved in the study. Data collected from each cluster were pooled to make inferences with respect to children under-5 and standard errors were adjusted for design effects for using the cluster sampling approach.

### Data collection

A random computer-generated code was linked to each household. Members of each household were approached and those who provided consent were enrolled in the study. The baseline socio-demographic characteristics of household members and location (collected using Garmin eTrex® 30 GPS device) of households were collected and entered the mHIS system. At the health facility level, secondary data were collected for the purposes of validation of the health status of children enrolled. The mHIS was implemented only in the intervention site, however, data were collected from both intervention and control sites.

Data were collected and aggregated from caregivers residing within the geographically accepted boundaries of the project. Caregivers were given guidance on the use of the mHis system by research assistants.

All households in the various communities were enumerated. A non-gender preference approach which seeks to give equal opportunities to caregivers, irrespective of gender, was used to collect socio-demographic data on children under-5 years and their caregivers, including age of child(ren) under-5, age of caregiver, marital status, level of education ethnic group and phone numbers. A unique code was generated and assigned to all children included in the study. Furthermore, caregivers were asked about symptoms for the childhood illnesses under review and given further guidance on identifying danger signs in children under-5 when necessary to ensure accuracy in reporting.

We assessed the primary endpoint, incidence of childhood illness (malaria, cough and diarrhoea) at baseline and endline, by asking the caregivers whether the child had experienced any of the common childhood illnesses in the last six (6) months during the time of interview. This information was verified for all children included in the study who were taken to a health facility. Health records were reviewed to confirm the diagnoses, with malaria confirmed through either rapid diagnostic test (RDT) or microscopy. Diarrhoea was defined as the occurrence of three or more loose or watery stools within a single day. Additionally, cases of cough were also recorded and used as proxy to calculating the incidence of Acute Respiratory Infections. The total number of confirmed cases of malaria, diarrhoea and cough within the study period were obtained from patient folders. This was used in calculating the incidence with reference to the total number of children under-5 (population at risk).

### Data management and analysis

Data was captured using CommCare (a leading software platform for field data capture) and transported to STATA 16 for data analysis. CommCare is a smartphone-based system that assisted in recording real-time information during home visits. We used Chi-square/Fisher’s exact test to examine the pre-existing differences between the intervention and control group with regard to the baseline sociodemographic characteristics of the caregivers and the children, with *p*-values < 0.05 considered statistically significant. To determine the differences between those who received the intervention and those who did not, the Average Treatment Effect on the Treated (ATET) was computed to show the average impact of the intervention.

### Ethics

Ethical clearance for the study was obtained from the Kwame Nkrumah University of Science and Technology (KNUST)/Komfo Anokye Teaching Hospital (KATH) Committee on Human Research, Publications and Ethics (CHRP/12/23). In addition, clearance and permission was obtained from the Director Health Administration of the Asante Akyim North Municipal District. Permission from the respective leaders of the communities also sought. Written informed consent was obtained from parents/legal guardians and assent from parents of caregivers less than 16 years of age.

## Results

### Child, caregiver, and household characteristics

Table 1 shows that there was a small difference in the mean age of children in the intervention and control groups, with values of 1.8 and 1.9 years respectively (*p* = 0.049). Gender distribution showed no significant difference between groups, with 49.3% males in the intervention and 50.7% in the control group (*p* = 0.499). At baseline, children in the intervention group had higher reported cases of diarrhoea (14.9%) compared to the control group (12.9%), though this difference was not statistically significant (*p* = 0.131). In contrast, the prevalence of cough at baseline was higher in the control group as compared to the intervention group (*p* < 0.001). Similarly, malaria prevalence was significantly lower in the intervention group (17.3%) compared to the control group (23.9%) (*p* < 0.001).

**Table 1:** Child, caregiver, and household characteristics

Variable	Intervention n (%)	Control n (%)	Total n (%)	Chi-square/fisher’s exact
*Child*				
Age (years), mean (SD)	1.8 (0.1)	1.9 (0.1)	1.9 (0.1)	0.049
Male gender	628 (49.3)	635 (50.7)	1263 (50.0)	0.499
Has diarrhoea	190 (14.9)	161 (12.9)	351 (13.9)	0.131
Has coughing	222 (17.4)	251 (20.0)	473 (18.7)	< 0.001
Has presumptive malaria (fever)	220 (17.3)	299 (23.9)	519 (20.6)	< 0.001
*Caregiver*				
Age (years), mean (SD)	30.4 (0.3)	34.2 (0.4)	32.1 (0.2)	< 0.001
Female gender	932 (91.4)	770 (86.8)	1702 (89.3)	0.001
Marital status				< 0.001
Cohabitation	287 (28.1)	183 (20.6)	470 (24.7)	
Married	604 (59.2)	550 (62.0)	1154 (60.5)	
Single	45 (4,4)	58 (6.5)	103 (5.4))	
Divorce/separated/widowed	77 (7.6)	94 (10.6)	171 (9.0)	
Education level				< 0.001
No formal education	176 (17.3)	152 (17.4)	328 (17.2)	
Primary/JSS	668 (65.5)	633 (71.4)	1301 (68.2)	
Secondary	137 (13.4)	66 (7.4)	203 (10.6)	
Postsecondary	39 (3.8)	36 (4.1)	75 (3.9)	
Knows diarrhoea is preventable	180 (17.7)	278 (32.3)	458 (24.3)	< 0.001
Knows signs of pneumonia	76 (7.5)	131 (15.2)	207 (11.0)	< 0.001
Knows causes of malnutrition	150 (14.7)	336 (39.0)	486 (25.2)	< 0.001
Knows malaria prevention	469 (46.0)	488 (55.0)	957 (50.2)	< 0.001
Knows signs of severe malaria	927 (90.9)	845 (95.3)	1772 (92.9)	< 0.001
*Household characteristics*				
Has improved source of drinking water	773 (87.9)	688 (89.0)	1461	0.121
Has improved sanitation facility	865 (98.4)	721 (93.4)	1586 (96.1)	< 0.001
Uses clean fuel for cooking	47 (5.4)	32 (4.2)	79 (4,8)	0.257

Significant differences were observed in caregiver characteristics. Caregivers in the intervention group were younger on average (mean age = 30.4, SD = 0.3) compared to those in the control group (mean age = 34.2, SD = 0.4). The gender distribution of caregivers showed a statistically significant difference (*p* = 0.001), with 91.4% females in the intervention group and 86.8% in the control group. Marital status exhibited notable distinctions (*p* < 0.001), with a higher proportion of cohabitation among intervention caregivers (28.1%) compared to control caregivers (20.6%).

**Fig. 1:**
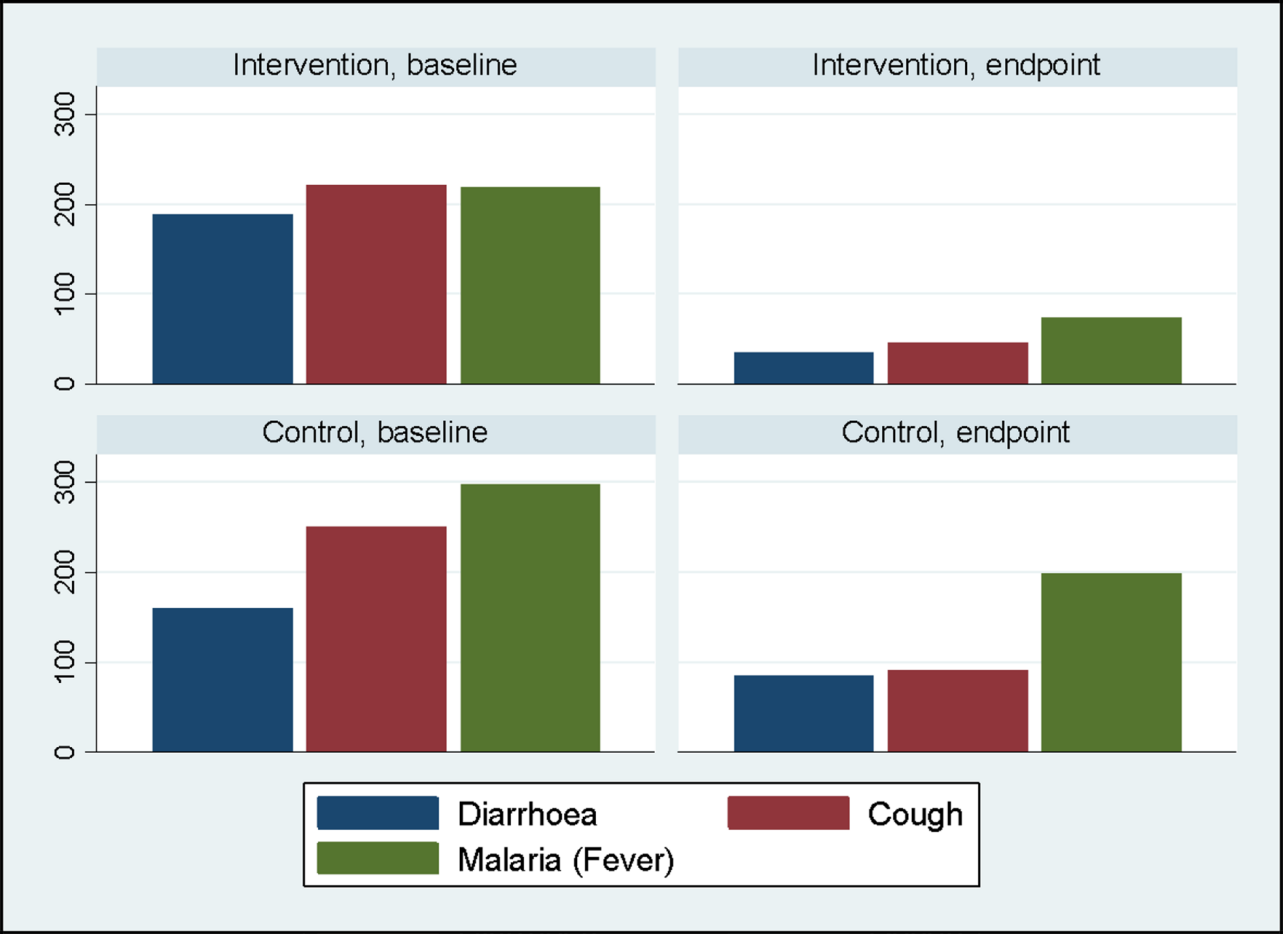
A graph of the outcomes (childhood illness) in the intervention group and the control group at baseline and at the endpoint

In Fig. 1, educational disparities were also evident (*p *< 0.001), with the intervention group having a higher percentage of individuals with secondary education (13.4%) compared to the control group (7.4%). However, at baseline, caregivers in the intervention group demonstrated a lower awareness of preventive measures, including knowledge about the preventability of diarrhoea (17.7% in intervention vs. 32.3% in control, *p* < 0.001), signs of pneumonia (7.5% in intervention vs. 15.2% in control, *p* < 0.001), causes of malnutrition (14.7% in intervention vs. 39.0% in control, *p* < 0.001), prevention of malaria (46.0% in intervention vs. 55.0% in control, *p* < 0.001), and signs of severe malaria (90.9% in intervention vs. 95.3% in control, *p* < 0.001). Regarding household characteristics, no significant differences were observed in improved drinking water sources (87.9% in intervention vs. 89.0% in control, *p* = 0.121) and the use of clean fuel for cooking (5.4% in intervention vs. 4.2% in control, *p* = 0.257). However, a substantial distinction was noted in the presence of improved sanitation facilities, with 98.4% in the intervention group and 93.4% in the control group (*p* < 0.001).

### Incidence of childhood illness at baseline and endpoint

Figure 1 illustrates the impact of the various outcomes at both baseline and endpoint. Across the board, there was a consistent decline in the reported outcomes of interest following the post-intervention period. Notably, when comparing the intervention and control groups, the reduction observed in the intervention group was more substantial than that in the control group at the endpoint. Specifically, in the intervention group, reported cases of diarrhoea decreased from 190 children at baseline to 30 children at the endpoint, cases of cough reduced from 220 to 47, and malaria cases decreased from 220 to 75. In the control group, reported cases of diarrhoea decreased from 161 children at baseline to 86 at the endpoint, cases of cough reduced from 251 at baseline to 92 at the endpoint, and malaria cases decreased from 299 at baseline to 199 at the endpoint.

Table 2 shows the incidence rate ratios (IRR) for the three primary health outcomes—Diarrhoea, Cough, and Malaria—examined at both baseline and endpoint periods. At baseline, the IRR for diarrhoea and cough stood at 1.16 and 0.87 respectively, although this was not statistically significant in both cases (95% CI 0.93–1.44 and 0.72–1.04). However, at the endpoint, a noteworthy reduction in diarrhoea occurred, with the IRR reducing to 0.56 (95% CI 0.38–0.86), signifying a reduction in the incidence of diarrhoea in the intervention group. Similar to the IRR at baseline for cough, IRR at endpoint was not statistically significant (IRR = 0.71, 95% CI 0.49–1.01). The baseline IRR for malaria was 0.72 (95% CI 0.61–0.86), the IRR then plummeted to 0.52 (95% CI 0.39–0.68) at the endpoint.

**Table 2:** Incidence rate ratio (IRR) for childhood illness for the intervention group and the control group at baseline and endpoint

Outcome	Baseline	Endpoint
	IRR (95% CI)	IRR (95% CI)
Diarrhoea	1.16 (0.93–1.44)	0.56 (0.38–0.86)
Cough	0.87 (0.72–1.04)	0.71 (0.49–1.01)
Malaria	0.72 (0.61–0.86)	0.52 (0.39–0.68)

### The difference in proportion of outcomes (childhood illness) by the intervention and the control groups at baseline and at endpoint

Table 3 presents a comparison between the intervention and control groups at both baseline and post-intervention for each specific outcome. At baseline, 14.3% of children in the intervention group reported diarrhoea, and this proportion decreased to 6.6% post-intervention. Therefore, a significant reduction in the proportion of diarrhoea was observed in the intervention group (diff = 0.032, 95% CI 0.004–0.060). In the control group, baseline diarrhoea cases were 12.9%, decreasing slightly to 11.5% at the endpoint, but the difference in proportion was not significant. For cough, there was a reduction from 17.4% in the intervention group at baseline to 8.6% post-intervention. In the control group, the prevalence changed from 20.0 to 12.3%. A significant difference in proportion was observed for both the intervention group (diff = 0.087, 95% CI 0.056–0.119) and the control group (diff = 0.087, 95% CI 0.053–0.122). Regarding presumptive malaria (fever), the intervention group experienced a reduction in malaria cases, dropping from 17.3% at baseline to 13.7% post-intervention. In the control group, reported cases increased from 23.9 to 26.6%. The differences in the proportion of malaria cases in the intervention group and the control group were not found to be significant.

**Table 3:** A comparison of intervention and control group by each outcome at baseline and postintervention

Outcome	Intervention			Control		
	Baseline n (%)	Endline n (%)	Diff (95% CI)	Baseline n (%)	Endline n (%)	Diff (95% CI)
*Diarrhoea*			0.032 (0.004–0.060) *			0.003 (−0.026 to 0.032)
Yes	190 (14.3)	36 (6.6)		161 (12.9)	86 (11.5)	
No	1083 (85.1)	513 (93.4)		1092 (87.2)	661 (88.5)	
Total	1273 (100)	549 (100)		1253 (100)	747 (100)	
*Cough*			0.087 (0.056 to 0.119) ***			0.087 (0.053–0.122) ***
Yes	222 (17.4)	47 (8.6)		251 (20.0)	92 (12.3)	
No	972 (81.4)	493 (89.8)		966 (77.1)	654 (87.6)	
Total	1194 (98.8)	540 (98.4)		1217 (97.1)	746 (99.9)	
*Malaria (fever)*			0.006 (0.031 to 0.042)			−0.009 (0.051 to 0.033)
Yes	220 (17.3)	75 (13.7)		299 (23.9)	199 (26.6)	
No	966 (75.9)	464 (84.5)		937 (74.9)	547 (73.3)	
Total	1186 (93.2)	615 (98.2)		1236 (98.8)	746 (99.9)	

*Diff * Difference in mean proportion

*p < 0.05; **p < 0.01; ***p < 0.001

### Average treatment effects on the treated analysis and difference in differences estimation

In Table 4, the results of the average treatment effects on the treated (ATET) analysis are presented, focusing on the differences in the proportion of reported cases of various outcomes between the control and intervention groups at both baseline and endpoint, along with the computed differences in differences (DiD) estimates.

**Table 4:** Average treatment effects of mHIS on outcome (childhood illness) at baseline and at the endpoint

Outcome	Baseline				Endpoint				DiD
	Control	Intervention	Diff (BL)	SE	Control	Intervention	Diff (EL)	SE	
Diarrhoea	0.101	0.122	0.019	0.012	0.098	0.090	−0.049 ***	0.017	−0.069 ***
Cough	0.206	0.186	−0.020	0.015	0.119	0.099	−0.036	0.021	−0.016
Malaria (fever)	0.241	0.185	−0.055 ***	0.017	0.251	0.180	−0.126 ***	0.023	−0.073**

*Diff (BL)* Difference at Baseline, *Diff (EL)* Difference at Endline *DiD *Difference in Differences

***p < 0.01; **p < 0.05

The ATET is a key measure to understand effectiveness of an intervention, henceat baseline, the difference in the proportion of diarrhoea in the intervention group was 0.122, slightly higher than the control group's 0.101. For cough, the difference in proportion was 0.206 in the control group and 0.186 in the intervention group at the baseline. Malaria showed a difference in proportion in the control group to be 0.241 and 0.185 in the intervention group at baseline. However, there was a significant difference in the control and intervention groups for presumptive malaria (β: −0.054; 95% CI −0.086, −0.021) but not for diarrhoea (β = 0.019; 95% CI −0.0057, 0.0433) and cough (β: −0.020; 95% CI −0.052, 0.012) at baseline. At the endpoint, for diarrhoea, the intervention group showed a difference in proportion of 0.090, while the control group increased slightly to 0.098. For cough, at the endpoint, the difference in proportion was 0.099 in the intervention group and 0.119 in the control group. For malaria, the differences in proportion were 0.251 in the control group and 0.180 in the intervention group. ATET analysis at the endpoint showed disparities in the groups (control and intervention) for diarrhoea (β: −0.049; 95% CI −0.082, −0.016) and presumptive malaria (β: −0.126; 95% CI −0.171, −0.081) but not for cough (β: −0.036; 95% CI −0.077, 0.005). The DiD estimate demonstrated a significant difference of −0.049 (*p* < 0.01) for diarrhoea and −0.073 (*p* < 0.05) for malaria, indicating a reduction in diarrhoea and malaria cases postintervention. However, the DiD estimate for cough (−0.016, *p* > 0.05) was not statistically significant.

## Discussion

This study assessed the effect of mobile health (mHealth) interventions on childhood illness burden using a quasi-experimental study design. The mhealth intervention assessed in this paper was a mobile phone-based interactive voice response which was designed and implemented usinsg the RE-AIM framework: Reach, Effectiveness, Adoption, Implementation, and Maintenance.

The study demonstrated increased numbers of children free from diarrhoea and malaria, in the intervention group compared to controls, indicating successful reach of the intervention to improve child health outcomes. Additionally, the findings underscored mHealth interventions' potential in reducing childhood disease burden, consistent with prior research highlighting improvements in health outcomes such as disease reduction and medication adherence [8–10].

Initially, the low utilization of mobile health services among caregivers highlighted early adoption challenges. However, significant uptake in the intervention group throughout the study emphasized the importance of targeted strategies to enhance adoption rates. Qualitative findings on barriers to use of the mHealth system, published elsewhere [13] also reported early adoption challenges such as lack of direct access to mobile phones. The study also identified barriers such as limited access to mobile phones, network issues, and literacy challenges affecting the implementation of the Mobchild IVR mHealth system. Overcoming these barriers is crucial for optimizing intervention implementation and effectiveness.

This study's findings are consistent with past studies that have emphasized the relevance of mobile-based interventions in improving health outcomes such as disease burden reduction, retention, and medication adherence [14–17]. According to Fadil  et al. [14], the incidence of diarrhoea reduced in households on the mobile health intervention arm. In their study, increased knowledge about hand washing with soap and storage of treated water sent using interactive voice response was significantly associated with decreased incidence of diarrhoea. This finding was corroborated by George et al. [17], who also reported a significant drop in diarrhoea incidence after the introduction of a mobile-health intervention [18]. In addition, Soremekun et al. (2023), reported a decrease in the incidence of pneumonia and suspected [19]*.* These findings have significant implications for public health interventions aimed at reducing the incidence of diarrhoea, cough, and malaria.

One of the primary benefits of mHealth interventions is their ability to provide caregivers with timely and focused health information [20]. Caregivers gain essential knowledge about preventative measures, correct hygiene practices, and timely healthcare-seeking behaviours through instructional messaging and access to mobile applications. This gives caregivers the knowledge and skills they need to prevent common childhood diseases and respond appropriately when their children display symptoms. Most paediatric illnesses are accompanied by fever, and caregivers' rapid diagnosis and treatment of these symptoms is critical in ensuring children receive fast medical assistance.[21–23].

Some studies [8, 13] have previously demonstrated the positive effect of mobile phone-based education, which was also employed in this study. The dissemination of voice messages to caregivers in the intervention group, like in a previous study [13], could have potentially led to improvement in the level of knowledge of caregivers in the intervention site. This could have in effect also led to the reduction in the number of children who reported malaria and diarrhoea during the intervention period.

Furthermore, the majority of caregivers in the intervention group did not get health information via their mobile phones at the beginning of the study. As a result, there may have been a gap in the early acceptability of mobile health services, which highlights the necessity for targeted measures to increase caregiver utilization. However, the uptake of mobile health interventions showed a significant increase in the intervention group throughout the experiment. These findings emphasize the importance of considering the adoption and ongoing usage of mobile health interventions when assessing their overall impact. Maximizing caregiver involvement and continuing to implement mobile health interventions are crucial for children's long-term health. Understanding the factors that influence the adoption and sustainability of mobile health treatments might help healthcare practitioners build effective methods to increase their adoption among caregivers.

Despite its efficacy in lowering the burden of childhood illness and caregiver adoption, the MobChild IVR mHealth system encountered a few obstacles. The study discovered some difficulties that hampered the usage of the mobile health intervention system, including a lack of direct ownership and access to mobile phones, network issues, forgetting how to use the IVR system, and insufficient literacy abilities in utilizing mobile devices. Understanding the characteristics that influence the adoption and maintenance of mobile health treatments might help healthcare practitioners build effective methods to increase their uptake among caregivers. By overcoming these hurdles, healthcare interventions may be personalized to fit the needs and preferences of caregivers, encouraging increased engagement and long-term usage.

While this study shows that mHealth treatments are beneficial in lowering childhood illness incidence, there are certain caveats to consider. The study period may miss the long-term impacts, and further studies would be needed to determine the sustainability and long-term impact of mHealth interventions on child health outcomes. Furthermore, the study's generalizability may be restricted to the unique demographic and context in which it was done, and additional research in diverse settings is required to corroborate the findings. While demonstrating short-term efficacy, the study highlights the need for further research to assess long-term sustainability and impact of mHealth interventions on child health outcomes beyond the study period.

## Conclusion

This study has demonstrated the utility of mHealth interventions for reducing disease burden of key childhood diseases in the Asante-Akim Municipality, Ghana. These findings show that mobile health interventions have the potential to improve child health outcomes by providing carers with specialist health information and guiding them to prevent and manage childhood diseases.

However, in order to increase the adoption and continued use of mobile health treatments, barriers such as network difficulties, a lack of direct ownership and access to mobile phones, and inadequate literacy skills must be addressed.

## Data Availability

The data for this study are available upon formal request from the corresponding author’s institution.
